# Cross-scanner and cross-protocol diffusion MRI data harmonisation: A benchmark database and evaluation of algorithms

**DOI:** 10.1016/j.neuroimage.2019.01.077

**Published:** 2019-07-15

**Authors:** Chantal MW. Tax, Francesco Grussu, Enrico Kaden, Lipeng Ning, Umesh Rudrapatna, C. John Evans, Samuel St-Jean, Alexander Leemans, Simon Koppers, Dorit Merhof, Aurobrata Ghosh, Ryutaro Tanno, Daniel C. Alexander, Stefano Zappalà, Cyril Charron, Slawomir Kusmia, David EJ. Linden, Derek K. Jones, Jelle Veraart

**Affiliations:** a Cardiff University Brain Research Imaging Centre (CUBRIC), School of Psychology, Cardiff University, Cardiff, United Kingdom; b Queen Square MS Centre, UCL Queen Square Institute of Neurology, Faculty of Brain Sciences, University College London, London, United Kingdom; c Centre for Medical Image Computing, Department of Computer Science, University College London, London, United Kingdom; d Harvard Medical School, Boston, MA, United States; e Image Sciences Institute, Department of Radiology, University Medical Center Utrecht, Utrecht, the Netherlands; f Department of Radiology, University of Pennsylvania and the Children's Hospital of Philadelphia, Philadelphia, PA, United States; g Institute of Imaging & Computer Vision, RWTH Aachen University, Aachen, Germany; h Machine Intelligence and Perception Group, Microsoft Research Cambridge, Cambridge, United Kingdom; i Mary McKillop Institute for Health Research, Australian Catholic University, Melbourne, Australia; j New York University, New York, NY, United States; k imec-Vision Lab, Department of Physics, University of Antwerp, Antwerp, Belgium

## Abstract

Diffusion MRI is being used increasingly in studies of the brain and other parts of the body for its ability to provide quantitative measures that are sensitive to changes in tissue microstructure. However, inter-scanner and inter-protocol differences are known to induce significant measurement variability, which in turn jeopardises the ability to obtain ‘truly quantitative measures’ and challenges the reliable combination of different datasets. Combining datasets from different scanners and/or acquired at different time points could dramatically increase the statistical power of clinical studies, and facilitate multi-centre research. Even though careful harmonisation of acquisition parameters can reduce variability, inter-protocol differences become almost inevitable with improvements in hardware and sequence design over time, even within a site. In this work, we present a benchmark diffusion MRI database of the same subjects acquired on three distinct scanners with different maximum gradient strength (40, 80, and 300 mT/m), and with ‘standard’ and ‘state-of-the-art’ protocols, where the latter have higher spatial and angular resolution. The dataset serves as a useful testbed for method development in cross-scanner/cross-protocol diffusion MRI harmonisation and quality enhancement. Using the database, we compare the performance of five different methods for estimating mappings between the scanners and protocols. The results show that cross-scanner harmonisation of single-shell diffusion data sets can reduce the variability between scanners, and highlight the promises and shortcomings of today's data harmonisation techniques.

## Highlights

• A benchmarking database for diffusion MRI data harmonisation is presented.
• The same 14 healthy controls were scanned on 3 scanners with 5 acquisition protocols.
• 5 harmonisation algorithms are compared for two tasks.
• 1) matched resolution scanner-to-scanner mapping, 2) spatial and angular resolution enhancement.
• Cross-scanner harmonisation can reduce the variability between scanners and protocols.

## 1 Introduction

Diffusion-weighted magnetic resonance imaging (dMRI) is being used increasingly to characterise tissue microstructure in health and disease (Johansen-Berg and Behrens, 2009; Jones, 2010a). Despite the promise of dMRI providing quantitative measures related to tissue microstructure, an inherent variability exists in the measurements when the same experiment is repeated on different scanners or at different time points. This inter- and intra-scanner variability can be caused by various factors including, but not limited to, differences in field strength, maximum available gradient strength, reconstruction technique from k-space data, positioning of the participant, imaging gradient non-linearities, number and sensitivity of the receiver coils, software versions used, and changes in the system calibration (Mirzaalian et al., 2016).

In addition to scanner-related variations, differences in acquisition protocol parameters introduce an extra source of variability in the measurements (Jones, 2010b). For example, even though guidelines for diffusion tensor MR imaging (DT-MRI) acquisitions have been proposed (e.g., Jones and Leemans, 2011), a standardised protocol is currently missing, i.e. the number and distribution of diffusion gradient directions in clinical research protocols tend to vary across sites. Nevertheless, protocol differences in studies that involve multiple scanners and/or are done over long periods of time are sometimes inevitable and not necessarily a sign of suboptimal experiment design. For example, developments of biophysical models increased the adoption of multiple b-value diffusion acquisition protocols over time, which concomitantly further increases the degrees of freedom in protocol design. Moreover, with technical advances in scanner hardware and software, protocol-updates during a study become desirable because they allow investigators to exploit such technical improvements in data acquisition. Notably, simultaneous multislice imaging (Feinberg et al., 2010; Larkman et al., 2001; Nunes et al., 2006) allows for the acquisition of more image volumes per unit time, and stronger-gradient systems (Jones et al., 2018; Setsompop et al., 2013) facilitate the acquisition of higher SNR data per unit time because of the reduced echo time (TE), where a trade-off can be made with smaller voxel-sizes and/or higher b-values.

Notwithstanding the challenges introduced by cross-scanner and cross-protocol differences, in the current era of “big data” there is strong interest in reliable combination of data acquired on different MRI scanners and/or with different protocols. Combining data from different scanners could increase the statistical power and sensitivity of studies, with obvious benefits in trials and multi-centre research, particularly in rare diseases or with difficult-to-recruit participants. Combining data from different protocols and quality could enable the transfer of rich information content from state-of-the-art acquisitions (e.g. from specialized systems as the 300 mT/m gradient Connectom system (Setsompop et al., 2013)), to lower quality data, e.g. to enhance spatial or angular resolution, or to enhance features that are less pronounced in low b-value measurements (Alexander et al., 2017; Jones et al., 2018).

The process of finding a mapping between diffusion data sets acquired with different scanners or protocols and making them as comparable as possible has gained increased attention recently (Fortin et al., 2017; Karayumak et al., 2018; Mirzaalian et al., 2017, 2016; Pohl et al., 2016). Often, these approaches are evaluated on databases from different scanners where the subjects are matched for age, gender, handedness, and socio-economic status, such that no statistical differences are expected at the group level. Ideally, however, individual subjects would be rescanned on different systems in relatively quick succession, such that measurement differences can be clearly attributed to inter-scanner and/or inter-protocol differences. Such databases have been acquired in the context of testing the reproducibility of DT-MRI metrics across scanners with different field strengths (1.5T vs 3T), sites, software versions, and vendors (Grech-Sollars et al., 2015; Vollmar et al., 2010; Zhu et al., 2011), but are (to the best of our knowledge) not publicly available.

Here, we present a benchmarking database of human brains that provides a testbed for data harmonisation^1^ across 3 different scanners and 5 different acquisition protocols, along with a comparison of 5 dMRI harmonisation algorithms. The database consists of acquisitions of the same 14 healthy participants scanned on MR systems with different maximum gradient strength (40, 80, and 300 mT/m). On the 80 mT/m and 300 mT/m systems, two types of protocols were acquired: 1) a ‘standard’ protocol with acquisition parameters matched as closely as possible to those on the 40 mT/m system (i.e. a typical clinical protocol); and 2) a ‘state-of-the-art’ protocol where the superior hardware and software specifications were utilised to increase the number of acquisitions and spatial resolution per unit time. In a recent open competition^2^, entrants were invited to implement and optimise algorithms that would harmonise data collected under these different scenarios. The algorithms are evaluated based on their performance in two tasks: 1) matched resolution scanner-to-scanner mapping between the standard acquisitions; and 2) spatial and angular resolution enhancement, finding a mapping between the standard acquisition of the 40 mT/m system to the state-of-the-art acquisition of the other systems.

## 2 Methods

The database and acquisition parameters for the three different scanners are described in detail in Section 2.1. Section 2.2 describes the harmonisation tasks that were formulated for the open competition where entrants were invited to evaluate their algorithms on the database. To minimise confounding effects of differences in preprocessing between different harmonisation algorithms during evaluation, we provided a minimally processed version of the database (described in Section 2.3). Section 2.4 describes the strategy used to evaluate algorithm outputs, and Section 2.5 summarises the algorithms proposed for scanner-to-scanner mapping and image quality enhancement to solve the proposed inter-scanner mapping tasks.

### 2.1 Data

14 healthy volunteers were included in the study (10 females, average age 25.7 years with range 21–41 years, Table 1), which was approved by Cardiff University School of Psychology ethics committee. Written informed consent was obtained from all subjects. The same 14 subjects were scanned on three different 3T scanners with different maximum gradient strengths: a) 3T GE Signa Excite HDx (40 mT/m), b) 3T Siemens Prisma (80 mT/m), and c) 3T Siemens Connectom (300 mT/m). The average time between acquisitions on scanners a) and b), and a) and c) was 21 and 22 months, respectively. The scanners had no software upgrades during the course of the study.

**Table 1:** Healthy volunteers included in the study.

Subject	Age^a^	Gender	Time gap scans a) and b) [months]	Time gap scans a) and c) [months]
**A**	26	f	14	16
**B**	21	m	23	24
**C**	41	f	14	16
**D**	25	f	23	23
**E**	21	f	21	21
**F**	25	f	28	30
**G**	25	f	28	28
**H**	28	f	20	20
**I**	21	m	21	21
**J**	22	f	23	23
**K**	26	m	28	30
**L**	35	m	16	16
**M**	23	f	23	23
**N**	21	f	20	20

a Age at the time of the first scan.

Spin-echo echo-planar dMRI images (SE-EPI) were acquired with a ‘standard’ (ST) protocol on all three scanners, and a ‘state-of-the-art’ (SA) protocol on the 80 mT/m and 300 mT/m systems (Table 2, Fig. 1). For the SA protocol, we exploited multiband-acquisition and the stronger gradients to shorten TE and improve the spatial- and angular resolution per unit time. Additional b = 0 s/mm^2^ images were acquired with TE and/or TR matching between protocols. Magnitude data was obtained for all scanners and protocols, and phase data was additionally saved for the 80 mT/m and 300 mT/m systems. Further information on the estimated SNR (Veraart et al., 2016b) is reported in Supplementary Table 1. Structural MPRAGEs (Magnetization Prepared RApid Gradient Echo) (de Lange et al., 1991) were acquired for each scanner and subject. The data is available for researchers upon request (see Section 4.5).

**Table 2:** Acquisition parameters for the different scanners and protocols.

Scanner	GE 40 mT/m	Siemens 80 mT/m		Siemens 300 mT/m	
Protocol	Standard (ST)	Standard (ST)	State-of-the-art (SA)	Standard (ST)	State-of-the-art (SA)
**Diffusion weighted images**					
Sequence	TRSE	PGSE	PGSE	PGSE	PGSE
b-values [s/mm^2^]	1200	1200, 3000	1200, 3000, 5000	1200, 3000	1200, 3000, 5000
# directions per b-value	30	30	60	30	60
TE [ms]	89	89	80	89	68
TR [ms]	Cardiac gated	7200	4500	7200	5400
Δ/δ [ms]		41.4/26.0	38.3/19.5	41.8/28.5	31.1/8.5
$\delta_{1}=\delta_{4}$/$\delta_{2}=\delta_{3}$ [ms]	11.23/17.84				
Phase encoding direction	AP	AP	AP	AP	AP
Acquired voxel size [mm^3^]	2.4 × 2.4 × 2.4	2.4 × 2.4 × 2.4	1.5 × 1.5 × 1.5	2.4 × 2.4 × 2.4	1.2 × 1.2 × 1.2
Reconstructed voxel size	1.8 × 1.8 × 2.4	1.8 × 1.8 × 2.4	1.5 × 1.5 × 1.5	1.8 × 1.8 × 2.4	1.2 × 1.2 × 1.2
Matrix size	96 × 96	96 × 96	154 × 154	96 × 96	180 × 180
# slices	60	60	84	60	90^a^
SMS factor	1	1	3	1	2
Parallel imaging	ASSET 2	GRAPPA 2	GRAPPA 2	GRAPPA 2	GRAPPA 2
Bandwidth [Hz/Px]	3906	2004	1476	2004	1544
Partial Fourier	5/6	–	6/8	6/8	6/8
Coil combine		Adaptive combine	Sum of Squares^b^	Adaptive combine	Adaptive combine
Head coil	8 channel	32 channel	32 channel	32 channel	32 channel
**b0 images**					
TE [ms]	89	89, 80, 89	80, 80, 89	89, 68, 89	68, 68, 89
TR [ms]	Cardiac gated	7200, 7200, 13000	4500, 7200, 7200	7200, 7200, 13000	5400, 7200, 7200
Phase encoding direction	AP	AP, PA	AP, PA	AP, PA	AP, PA

a A trade-off was made between number of slices and timing parameters, as a result the cerebellum was not always covered.

b This was the only strategy possible with the settings used. TRSE = twice-refocused spin-echo, PGSE = pulsed-gradient spin-echo.

**Fig. 1:**
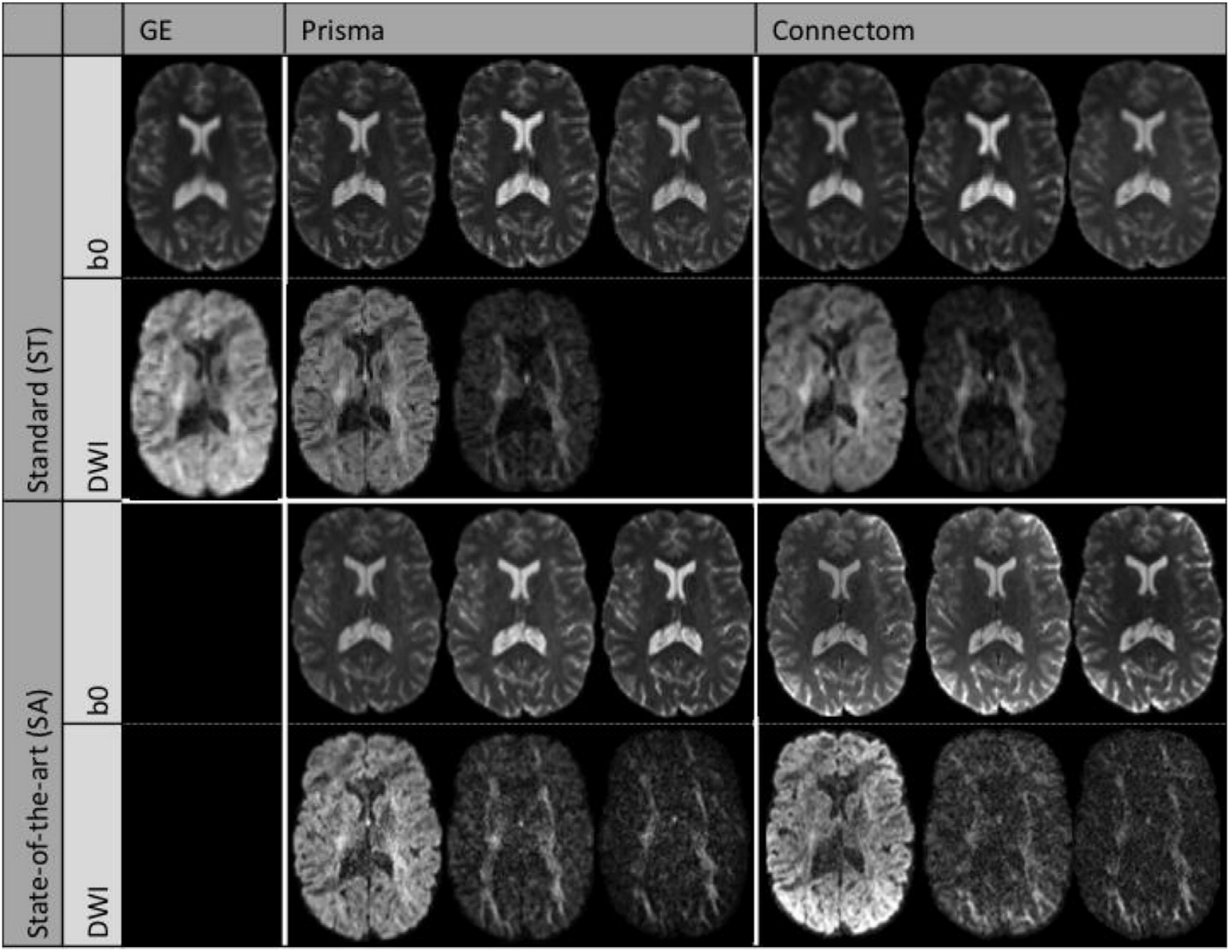
Example diffusion images of the ST and SA protocols from one subject after preprocessing. b0 images with three different combinations of TE/TR for the 80 mT/m and 300 mT/m scanners. DWIs with b-values (from left to right) 1200, 3000, and 5000 s/mm^2^.

### 2.2 Harmonisation tasks

The rich database, which includes images of the same subject acquired on different scanners with different b-values, angular resolutions, spatial resolutions, and timing parameters, allows for a wide variety of aspects to be evaluated. In this work, we focus on evaluating the process of finding a mapping between the lowest b-value shells across scanners and protocols. The b = 0 and 1200 s/mm^2^ data of all scanners and protocols from 10 randomly selected subjects (coined A-G, I-K in this study) were used as training dataset. Data from the remaining 4 subjects (H, L-N) were used as a testing set; the 40 mT/m data were distributed to the entrants of the challenge while the data of the other scanners were held back for further evaluation purposes.

Two tasks were evaluated:

1) Scanner-to-scanner mapping at matched resolution acquisition protocol, predicting the 80 mT/m and 300 mT/m ST signals in the cerebrum from the 40 mT/m ST signals; and
2) Spatial- and angular resolution enhancement, predicting the 80 mT/m and 300 mT/m SA signals in the cerebrum from the 40 mT/m ST signals.

### 2.3 Preprocessing

All datasets were manually checked for artifacts such as slice outliers, vibration artifacts, and interleave motion artifacts (Gallichan et al., 2009; Tax et al., 2016; Tournier et al., 2011). One DWI volume was excluded from one dataset (subject H of the test set). The data were subsequently preprocessed for each subject and scanner as detailed in the next paragraphs, where the steps were homogenised where possible and an additional gradient-nonlinearity distortion correction step was added for the 300 mT/m system. All data was spatially registered for each subject across scanners (with the mean DWI of the 80 mT/m ST acquisition as template), and the result was inspected manually.

The 40 mT/m data were corrected for eddy current distortions and subject motion with FSL EDDY (Andersson and Sotiropoulos, 2016) and corrected for EPI distortions by nonlinear registration of the mean of the DWIs to the 80 mT/m ST mean DWI with Elastix (Irfanoglu et al., 2015; Klein et al., 2010; Leemans et al., 2009) and b-matrix rotation (Leemans and Jones, 2009).

The 80 mT/m data were corrected for eddy current distortions, subject motion, and EPI distortions with FSL TOPUP (Andersson et al., 2003) and EDDY. The corrected 80 mT/m SA mean DWI was affinely registered to the 80 mT/m ST mean DWI with b-matrix rotation.

The 300 mT/m data were corrected for eddy current distortions, subject motion, EPI distortions, and gradient-nonlinearity distortions (Glasser et al., 2013) with FSL TOPUP and EDDY and in-house software kindly provided by Martinos Centre, Massachusetts General Hospital. The corrected 300 mT/m ST and SA mean DWI were affinely registered to the 80 mT/m ST mean DWI with b-matrix rotation.

The MPRAGE of each scanner was affinely registered to the 80 mT/m ST mean DWI. Face removal was subsequently performed (Bischoff-Grethe et al., 2007). Brain masks excluding the cerebellum were obtained from the MNI atlas, by warping the mask in MNI space non-linearly to each subject's DWI space with a repeated call of FSL FNIRT and affine registration from FSL FLIRT used for initialisation (Andersson et al., 2007; Jenkinson et al., 2012).

### 2.4 Evaluation

Harmonisation algorithms had to predict the image matrix of the preprocessed 80 mT/m and 300 mT/m datasets by using the b-matrix files and the associated 40 mT/m image data of each of the four test subjects. From the *predicted* data of each test subject and each algorithm, the diffusion tensor was estimated using a weighted linear least squares estimator (Veraart et al., 2013) using the MRTrix software package, and fractional anisotropy (FA) and mean diffusivity (MD) were subsequently computed in each voxel. In addition, rotationally invariant spherical harmonic (RISH) features R0 and R2 (Mirzaalian et al., 2015) were computed after normalising the signal per voxel with the mean b = 0 signal to measure the angular frequency of the diffusion signals.

These results were evaluated against the ground truth features derived from the *acquired* data (Fig. 2). Different errors were computed to enable the characterisation of accuracy and precision: mean-error (ME, (predicted - acquired), measuring accuracy), mean-normalised error (MNE, (predicted - acquired)/acquired, measuring relative accuracy), and mean-squared error (MSE, (predicted - acquired)^2^, measuring accuracy and precision). The accuracy characterised by ME and MNE can be both positive and negative as this would indicate over/under-estimation of the metric, whereas MSE takes the absolute error into account. These errors were computed globally (in a brain mask), regionally (Freesurfer regions (Fischl et al., 2002)) and locally (sliding 3 × 3 × 3 voxels-neighbourhood). Voxels at the edge of the brain, the cerebellum, and systematically poor performing regions (see Section 3.1.2) were excluded. Further information on the number of brain voxels excluding the cerebellum is reported in Supplementary Table 2.

**Fig. 2:**
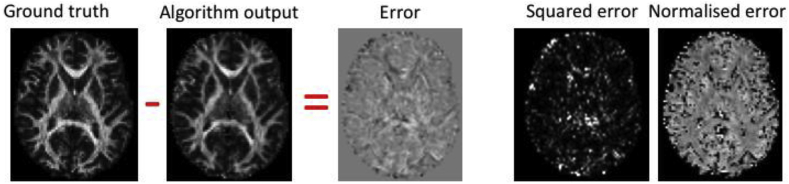
Evaluation procedure: the error is computed as the difference between the ground truth and predicted image, and from this the squared- and normalised error are computed.

### 2.5 Algorithms

Entrants to the open competition developed 5 different algorithms to solve the inter-scanner mapping tasks described in section 2.2 (task 1: scanner-to-scanner mapping of matched acquisition protocols; task 2: spatial/angular resolution enhancement). Additionally, a ‘reference’ prediction of the 80 mT/m and 300 mT/m ST and SA data was created from the 40 mT/m ST data using simple trilinear interpolation in the spatial domain, and spherical harmonics interpolation (order 6 for ST and 8 for SA) in the angular domain.

The algorithms developed by the entrants explored different deep learning architectures as well as dictionary learning techniques to solve the proposed tasks. They will be referred to as: spherical harmonic network (SHNet); spherical harmonic residual network (SHResNet); spherical network (SphericalNet); sparse dictionary learning (SDL) and fully convolutional shuffling network (FCSNet). A summary of the algorithms is presented in Table 3, a detailed description of each algorithm is provided in the subsections below.

**Table 3:** Summary of harmonisation algorithms evaluated.

Algorithm name	Additional preprocessing	Training domain	Core method	Algorithm details
SHNet	Brain extraction	SH	Deep learning	qDL inspired network, anatomically constrained training
SHResNet	Brain extraction	SH	Deep learning	Residual structure network, anatomically constrained training
SphericalNet	Brain extraction	SH	Deep learning	Local Spherical Convolution Network, anatomically constrained training
FCSNet	Brain extraction	SH	Deep learning	Fully convolutional network with task-dependent regularization (L2 and L1)
SDL		Over-complete data-driven dictionaries	Adaptive dictionary learning	Linear mapping between over-complete dictionaries

#### 2.5.1 Spherical harmonic network (SHNet)

The SHNet algorithm is a deep learning network inspired by elements of Golkov et al. (2015) and Koppers et al. (2017). In this algorithm, every signal was preprocessed by dividing by its baseline b = 0 measurement, followed by a conversion into the SH space (order four and Laplace-Beltrami regularization of λ=0.006). The network consisted of three fully connected layers with rectified linear units (ReLU) as activation function, followed by a batch normalization layer to stabilise the training process. An overview is given in Table 4.

**Table 4:** Topology of SHNet.

#Layer	Type	Parameters	Activation
1	Batch Normalization	–	–
2	Fully-Connected	From: #SH coefficients To: 150 neurons	ReLU
3	Batch Normalization	–	–
4	Fully-Connected	From: 150 neurons To: 150 neurons	ReLU
5	Batch Normalization	–	–
6	Fully-Connected	From: 150 neurons To: 150 neurons	ReLU
7	Batch Normalization	–	–
8	Fully-Connected	From: 150 neurons To: #SH coefficients	–

For training of the hyperparameters, 9 out of 10 subjects from the training set were used, with the remaining subject used for training validation before deployment on the test set of 4 additional subjects. During training, parameters were initialised using the Adam optimiser (learning rate of 0.001; batch size 128) (Kingma and Ba, 2014), which was replaced by the SGD optimiser (Robbins and Monro, 1951) after the first five epochs. Afterwards, the learning rate was decreased by ten percent if the performance did not improve for more than five epochs within the validation subject. The reduced learning rate leads to a better fine tuning of the network. Furthermore, training was only performed on voxels within a brain mask derived from FSL BET (Smith, 2002).

The SHNet described above was employed for task 1 (matched resolution scanner-to-scanner mapping). For task 2 (spatial/angular resolution enhancement), standard cubic interpolation is also utilised to increase the spatial resolution, while gradients are resampled utilising the predicted SH coefficients to deal with the increased higher angular resolution. The deep learning framework is based on PyTorch. The runtime per voxel on a Nvidia Geforce 1080Ti with 11 GB RAM was 6.4e-05 s.

#### 2.5.2 Spherical harmonic residual network (SHResNet)

The SHResNet algorithm (Koppers et al., 2018) is a deep learning network structure based on the novel concept of residual structure (He et al., 2016), which introduces a subtraction path from the input to the network output, resulting in very robust performance. Furthermore, the network focuses only on the difference between the input and its corresponding target signal. In addition, residual structures are efficiently trainable, even for very deep networks.

Data preprocessing utilised SH (order four and Laplace-Beltrami regularization of λ=0.006), while each signal was divided by its b = 0 measurement. In this network, three main 3D-convolutional layers (kernel size 3 × 3 × 3) processed the signal and predicted the difference for a specific harmonic order. The first two convolutional layers padded the signal to keep the spatial dimensions, while the last convolutional layer reduced the signal from a 3 × 3 × 3 voxel neighbourhood to a single voxel. Since each SH order was predicted separately, three individual networks were required for an SH order of four, which were combined with a fully connected layer. Afterwards, the resulting signal was subtracted from the corresponding input signal. A final fully connected layer was utilised to smooth the signal and to generate the predicted SH coefficients. An overview is given in Fig. 3.

**Fig. 3:**
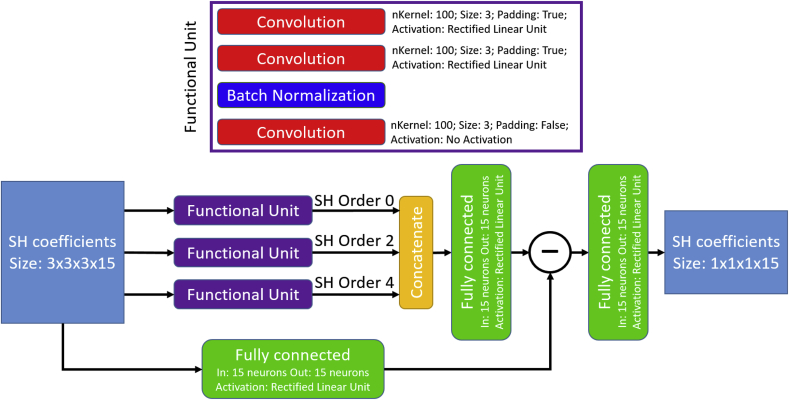
Structure of the SHRestNet.

Similarly to SHNet, SHResNet relied on 9 out of 10 subjects from the training set for the actual training, with the remaining subject used for training validation before deployment on the test set, while only voxel within a brain mask based on FSL BET (Smith, 2002) are considered for training. The network was initialised utilising the Adam optimiser (learning rate of 0.001; batch size 128) (Kingma and Ba, 2014), which is replaced by an SGD optimiser after the first five epochs. After this change, the learning rate was decreased by ten percent if the performance did not improve for more than five epochs.

The SHResNet described above was employed for task 1 (matched resolution scanner-to-scanner mapping). For task 2 (spatial/angular resolution enhancement), standard cubic interpolation is also utilised, as described for SHNet. The deep learning framework is based on PyTorch. The runtime per voxel on a Nvidia GeForce 1080Ti with 11 GB RAM was 0.0014 s.

#### 2.5.3 Spherical network (SphericalNet)

The SphericalNet algorithm utilises a novel deep learning structure based on spherical surface convolutions (Koppers and Merhof, 2018), which were designed especially for spherical signals. These convolutions utilise local gradient neighbourhood information to increase the accuracy of reconstruction, while spatial neighbourhood information is passed layer by layer. In the end, spatial information is combined within the last convolutional layer to project from a 3 × 3 × 3 voxel neighbourhood onto a 1 × 1 × 1 target voxel.

As preprocessing, every signal was transformed into the SH space to avoid a gradient-based mismatching, while the SphericalNet transformed every signal back into a predefined signal space, consisting of 30 equidistantly sampled gradient directions over a hemisphere. Afterwards, every voxel was processed by three spherical convolutions with a kernel size of one plus five and an angular distance of $\;\text{Θ}=\frac{\pi }{10}$. The angular distance defines the angle between the resulting (in this case five) sampled gradient directions and their corresponding main gradient direction. After each spherical convolution, sigmoid functions were used as activation functions to limit every signal's range between 0 and 1. Subsequently, every signal was converted back into SH space. A batch normalization layer normalised the resulting coefficients. In the end, three 3-D convolutional layers exploited additional spatial neighbourhood information with parametric rectified linear units (f(x)=kmax(0,x), with k being a learnable parameter) (PReLU) as activation functions. A complete overview of the network's architecture is given in Table 5.

**Table 5:** Architecture of the SphericalNet.

#Layer	Type	Parameters	Activation
1	Conversion	From SH to signal space (SH order 4; Laplace-Beltrami Regularization 0.006)	–
2	Spherical Surface Convolution (applied on gradient signals)	Input: 1 Shell; Output: 16 Shells; kernel size: 5; $\text{Θ}=\frac{\pi }{10}$	Sigmoid
3	Spherical Surface Convolution (applied on gradient signals)	Input: 16 Shell; Output: 16 Shells; kernel size: 5; $\text{Θ}=\frac{\pi }{10}$	Sigmoid
4	Spherical Surface Convolution applied on gradient signals)	Input: 16 Shell; Output: 16 Shells; kernel size: 5; $\text{Θ}=\frac{\pi }{10}$	Sigmoid
5	Conversion	From signal to SH space (SH order 4; Laplace-Beltrami Regularization 0.006)	
6	Batch Normalization	–	–
7	3D Spatial Convolution	Kernel size: 3 × 3 × 3, padding: 1	PReLU
8	3D Spatial Convolution	Kernel size: 3 × 3 × 3, padding: 1	PReLU
9	3D Spatial Convolution	Kernel size: 3 × 3 × 3, padding: 0	

The SphericalNet is trained on brain voxels (derived with FSL BET (Smith, 2002)) from 9 out of 10 training set subjects, with the remaining subject used for training validation before deployment on the test set. The network was initialised utilising the Adam optimiser (learning rate of 0.001; batch size 128) (Kingma and Ba, 2014), which is replaced by an SGD optimiser after the first five epochs. After this change, the learning rate was decreased by ten percent if the performance did not improve over five epochs to ensure a good fine tuning of the network.

The SphericalNet described above was employed for task 1 (scanner-to-scanner mapping). For task 2 (spatial/angular resolution enhancement), standard cubic interpolation is also utilised, as described for SHNet. The deep learning framework is based on PyTorch. The runtime per voxel on a Nvidia GeForce 1080Ti with 11 GB RAM was 0.0067 s.

#### 2.5.4 Fully-convolutional shuffling network (FCSNet)

The FCSNet algorithm relies on a patch-based fully-convolutional network (FCN) to solve matched resolution scanner-to-scanner harmonisation and resolution enhancement tasks as presented in this paper.

The FCSNet architecture was inspired by Tanno et al. (2017), and contained a “shuffle” operation in the last layer of a super-resolution network to efficiently compute a transpose-convolution as a final step (Shi et al., 2016). The FCSNet structure used here differed from the previous implementation in Tanno et al. (2017) as it contained four hidden layers and a skip connection (Fig. 4). Also, it relied on a different loss function (see below).

**Fig. 4:**
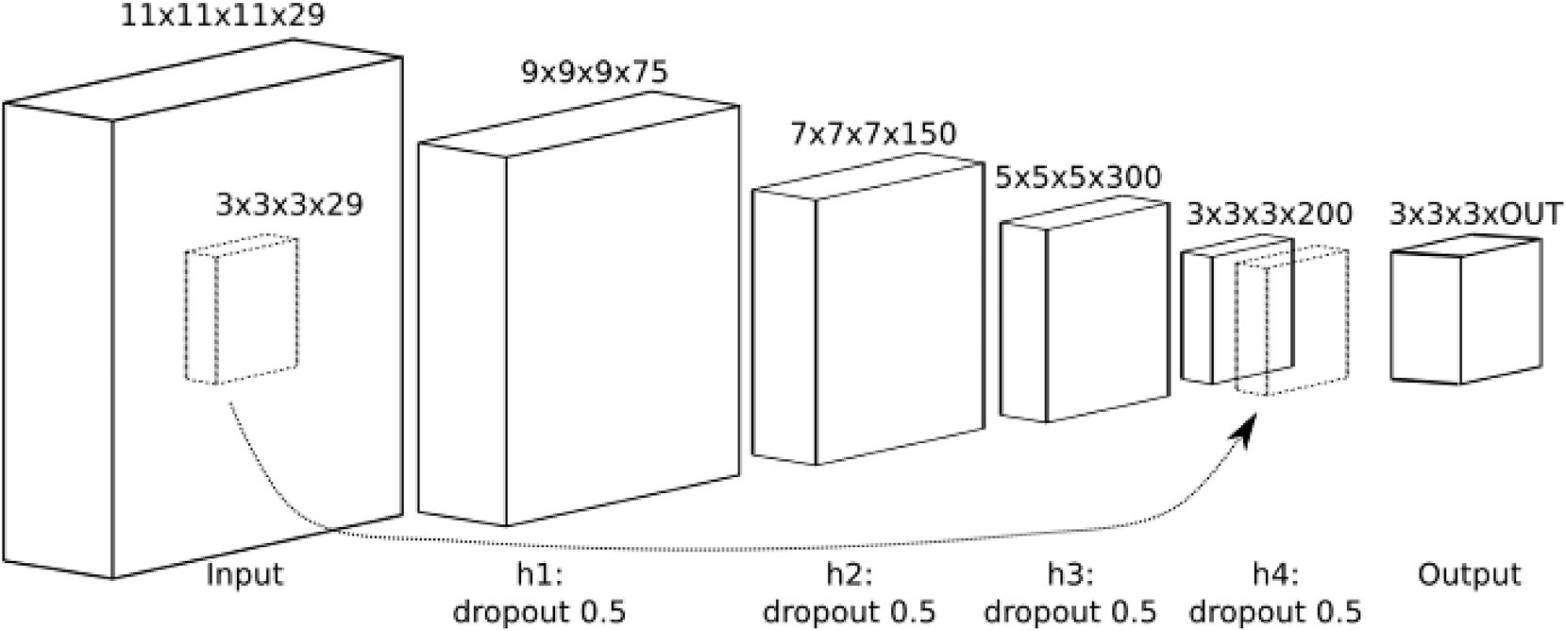
Architecture of the FCSNet with four hidden layers and a skip connection.

FCSNet processed SH coefficients obtained from signals within a brain mask, which was derived from FSL BET (Smith, 2002) and eroded to exclude boundary voxels with noisy signal. SH coefficients were estimated using Dipy (Garyfallidis et al., 2014), up to order 6 for ST protocols and up to order 8 for the SA protocols. For the actual training, SH coefficients were clipped to the 98th percentile of function values on the sphere over the masked image, as this further reduced noise.

For the training processes, sizes of 11 × 11 × 11 and of 3 × 3 × 3 were used for input and output patches respectively, with the number of input and output channels being 29 (input; SH order-6 plus b = 0) and 29 or 46 (output; SH order=6 or 8 plus b = 0). Each hidden layer consisted of a 3 × 3 × 3 convolution layer, a ReLu activation with varying filter lengths and a dropout layer with 0.5 keep probability. The last hidden layer had a skip-connection to the input layer to “sharpen” the prediction. The output layer was computed after a bottleneck convolution (see Fig. 4).

The loss function was constituted of two parts: a channel-wise loss, which gives equal weight to all channels and a loss on the function-value on the sphere, which enforces signal fidelity along hundred uniformly chosen directions on a hemisphere, while also considering the RISH constraints of order 0, 2, 4, 6 for the SA protocol (300 mT/m scanner).

At prediction time, the FCN was applied to juxtaposed input patches to fully cover the subject brains.

Hyperparameters (learning rate, number of layers and batch size) were selected based on the MSE on the validation set. The deep learning framework is based on TensorFlow. Training comprised of 200 epochs consisting of 50000 3D patch-pairs (input size: 11 × 11 × 11 × 29) selected randomly from the training images and evaluated using minibatches of size 20 and a learning rate of 1e-4. Training time was in the order of ten hours, while inference time for a masked brain (HCP resolution) is in the order of a minute. All training and testing were done on a server with 112 GB RAM with an NVIDIA GTX Titan X GPU.

#### 2.5.5 Sparse dictionary learning (SDL)

The SDL algorithm relies on the methodology recently developed in St-Jean et al. (2016, 2017), based on over-complete sparse dictionaries that are learned automatically from the data.

For harmonisation with SDL, N patches of small spatial and angular local neighbourhoods were extracted from all datasets and organised to create a set of column arrays $\left\{X_{1}\;,\;\ldots \;,\;X_{N}\right\}$, with each $X_{n}\in \;{\text{R}}^{m\times 1}$. Subsequently, sparse features were automatically created from the target scanner datasets using dictionary learning (Mairal et al., 2010). A sparse dictionary $D\in \;{\text{R}}^{m\times p}$ was found such that

(1) $$D^{*}=\arg{\text{min}}_{D}\sum_{n=1}^{N}||X_{n}-{\mathrm{D\alpha}}_{n}|{|}_{2}^{2}+\lambda ||\alpha_{n}|{|}_{1},s.t.||D|{|}_{2}^{2}=1,\alpha \geq 0,$$

with $\alpha_{n}\in \;{\text{R}}^{p\times 1}$ being an array of non-negative coefficients and D the dictionary initialised from patches randomly extracted from the datasets, set to have twice as many columns as rows (i.e. p=2m). Iterative updates alternating between refining **D** using eq. (1) (and holding α fixed) and updating α (with **D** held fixed) with a coordinate descent scheme (Friedman et al., 2010) were carried for 1000 iterations using a batchsize of 128 patches randomly sampled for each iteration. An automatic search for the regularization parameter λ was employed (Friedman et al., 2010). The search selected the value of λ according to the Akaike Information Criterion (AIC), where the number of non-zero elements in the dictionary was used as the number of degrees of freedom for the model (Tibshirani and Taylor, 2012).

For the reconstruction in task 1 (matched resolution scanner-to-scanner mapping), the dictionary was created using patches of size 3 × 3 × 3 × 5. Images were mean-subtracted and standardised to account for scaling, with the coefficients $\alpha_{n}$ subsequently unscaled afterwards. This idea assumes that there is a set of common features which can be mapped between acquisitions made on different scanners. The general reconstruction process is shown in Fig. 5.

**Fig. 5:**
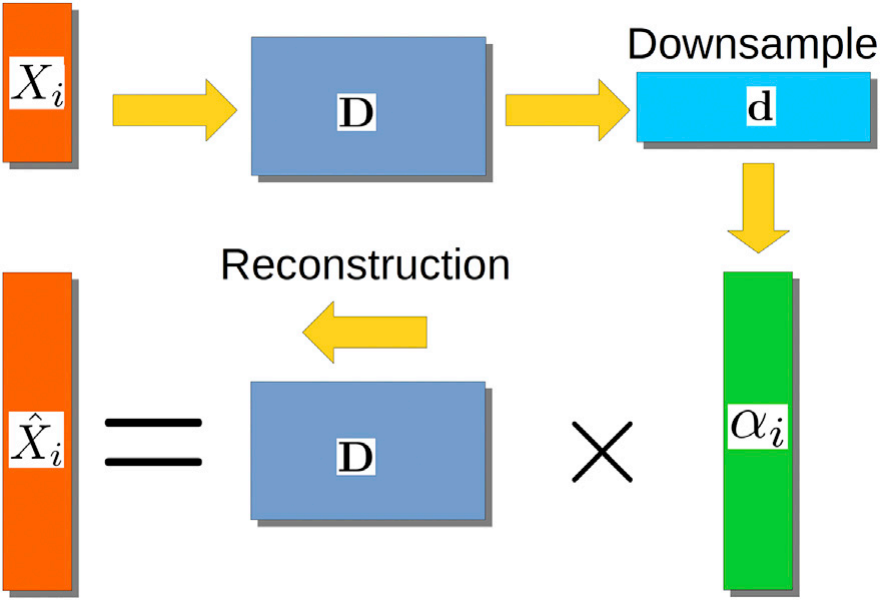
Reconstruction process for SDL. Local patches are decomposed into vectors **X**_i_ to build the dictionary **D**. From there, the coefficients $\alpha_{i}$ are computed from **D** for task no. 1 or using a downsampled version of **D** for task no. 2. The final reconstruction for each patch **X**_i_ is obtained by multiplying **D** and $\alpha_{i}$.

For the reconstruction in task 2 (spatial and angular resolution enhancement), patches of different spatial sizes were extracted from the images at lower resolution (ST protocol; patches of sizes 3 × 3 × 3) and from the images at higher resolution (SA protocol; patches of size 5 × 5 × 5 and 6 × 6 × 6), under the hypothesis that such sizes would yield a plausible representation between the lower resolution and higher resolution scans. Reconstruction coefficients $\alpha_{n}$ were computed on the downsampled dictionary and the final reconstruction used the original size dictionary (St-Jean et al., 2017). Finally, to match the gradient directions, the truncated SH basis of order 6 (Descoteaux et al., 2007) was used on each final dataset to predict the target images at the required gradient directions.

The training time for the matched resolution scanner-to-scanner mapping within a brain mask, 1000 epochs, was approximately 72 min on a quad cores Intel Xeon processor at 3.5 GHz with an average ram usage of around 600 MB. For predicting each dataset from the 40 mT/m scanner to the target scanner, it took approximately 4h30 min per dataset with an approximate ram usage of 315 MB.

## 3 Results

### 3.1 Matched resolution scanner-to-scanner mapping

#### 3.1.1 Global evaluation

Fig. 6 shows the global MSE of FA, MD, R0, and R2, respectively, i.e. the mean taken over all voxels within the mask. Results are shown for the 4 different test subjects. There does not seem to be a systematic deviation for one of the subjects, so all subjects were included for evaluation. Global MSE are shown for the different harmonisation approaches described in Section 2.5 (SHNet, SHResNet, SphericalNet, FCSNet, and SDL) along with the ‘reference’ prediction obtained from trilinear interpolation in the spatial domain and spherical harmonics interpolation in the angular domain.

**Fig. 6:**
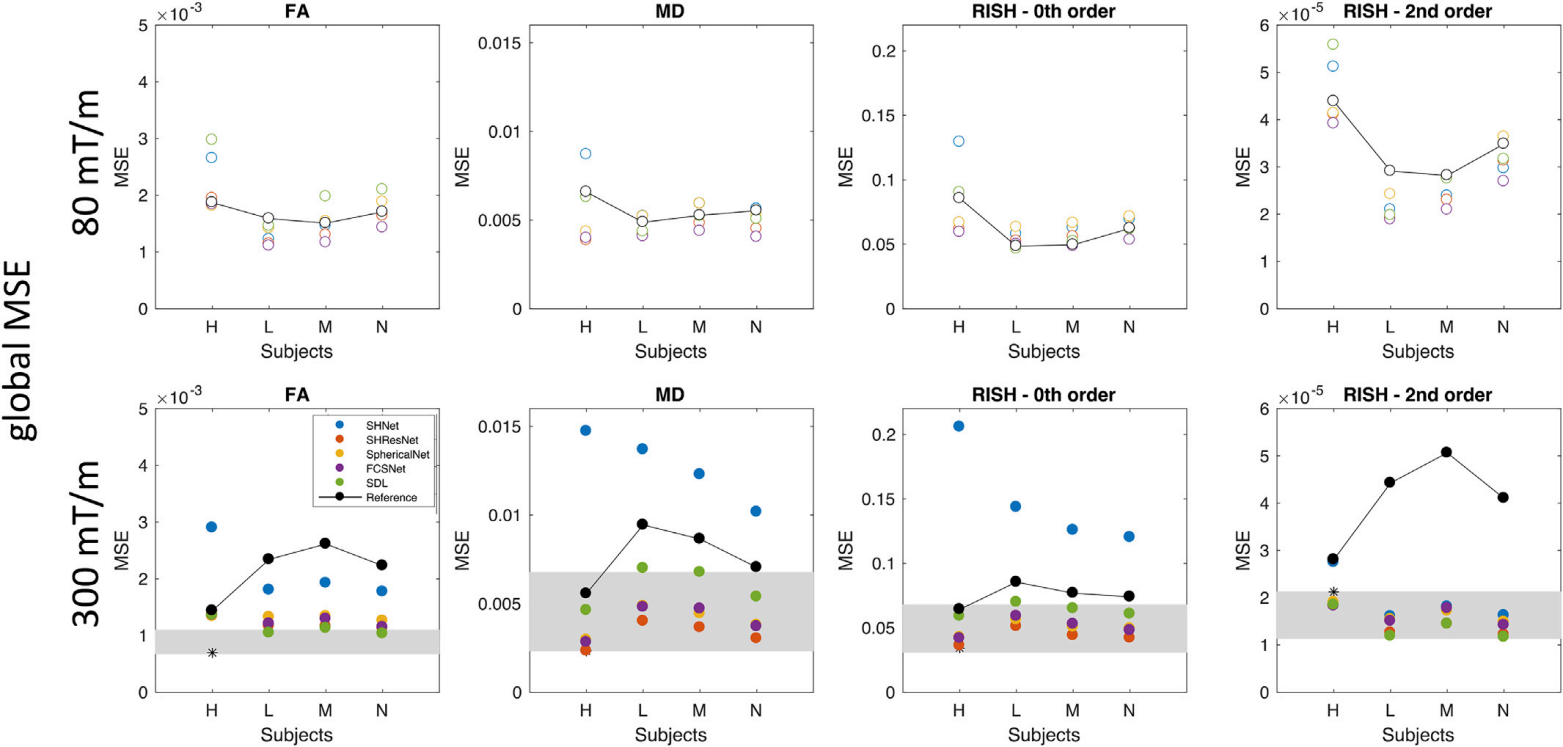
Results of the matched resolution scanner-to-scanner mapping: Global MSE for FA, MD, R0, and R2 (columns); for the 4 different test subjects; for predictions of the 80 mT/m data (top, open circles) and the 300 mT/m data (bottom, closed circles). The different methods are represented with different colours SHNet (blue), SHResNet (red), SphericalNet (orange), FCSNet (purple), SDL (green), and reference (black). The reference results for different subjects are connected with lines as a visual aid to compare with the performance of the proposed algorithms. The range of scan-rescan MSEs across three subjects is shown by the gray rectangle. The scan-rescan result for the only overlapping subject “H” is indicated by the *.

In most cases, the MSE for the harmonisation methods were lower than the reference. For FA, MD, and R2, this was true both for the 80 mT/m and 300 mT/m predictions. For R0, the MSE of the harmonisation methods was higher than that of the reference for the 80 mT/m prediction, except for subject H. For this subject, the SHNet 300 mT/m prediction has a consistently higher MSE than the reference for FA, MD, and R2. Fig. 6, bottom also shows the MSE of the submissions compared to scan-rescan differences, which provide the ultimate goal for data harmonisation. Scan-rescan data was only available for the 300 mT/m gradient system and for three subjects, of which one subject overlapped with the evaluated subjects, i.e. “H”. It can be observed that the best performing techniques approach the range of scan-rescan reproducibilities for all metrics, with an overall lower performance in terms of FA.

#### 3.1.2 Regional evaluation

Fig. 7 shows the mean-squared error for 143 different white and gray matter ROIs and all evaluated algorithms. The median MSE per ROI and per evaluated algorithm across subject is shown. The Freesurfer ROIs are labeled according to Supplementary Table 3. Some ROIs have a consistently higher error, possibly due to residual image misalignment between different scanners. Indeed, the following ROIs scored in the WM/GM-specific 90^th^ percentile for at least 3 algorithms, for at least one of the evaluated metrics: banks of the superior temporal sulcus, fusiform gyrus, temporal pole, transverse temporal gyrus, caudal anterior cingulate, insula, entorhinal, orbital part of inferior frontal gyrus, and frontal pole. However, no systematic trends in the major white matter bundles or the prefrontal cortex, where differences in susceptibility correction strategies could affect the result, were observed. Supplementary Table 3 marks systematically poor performing regions.

**Fig. 7:**
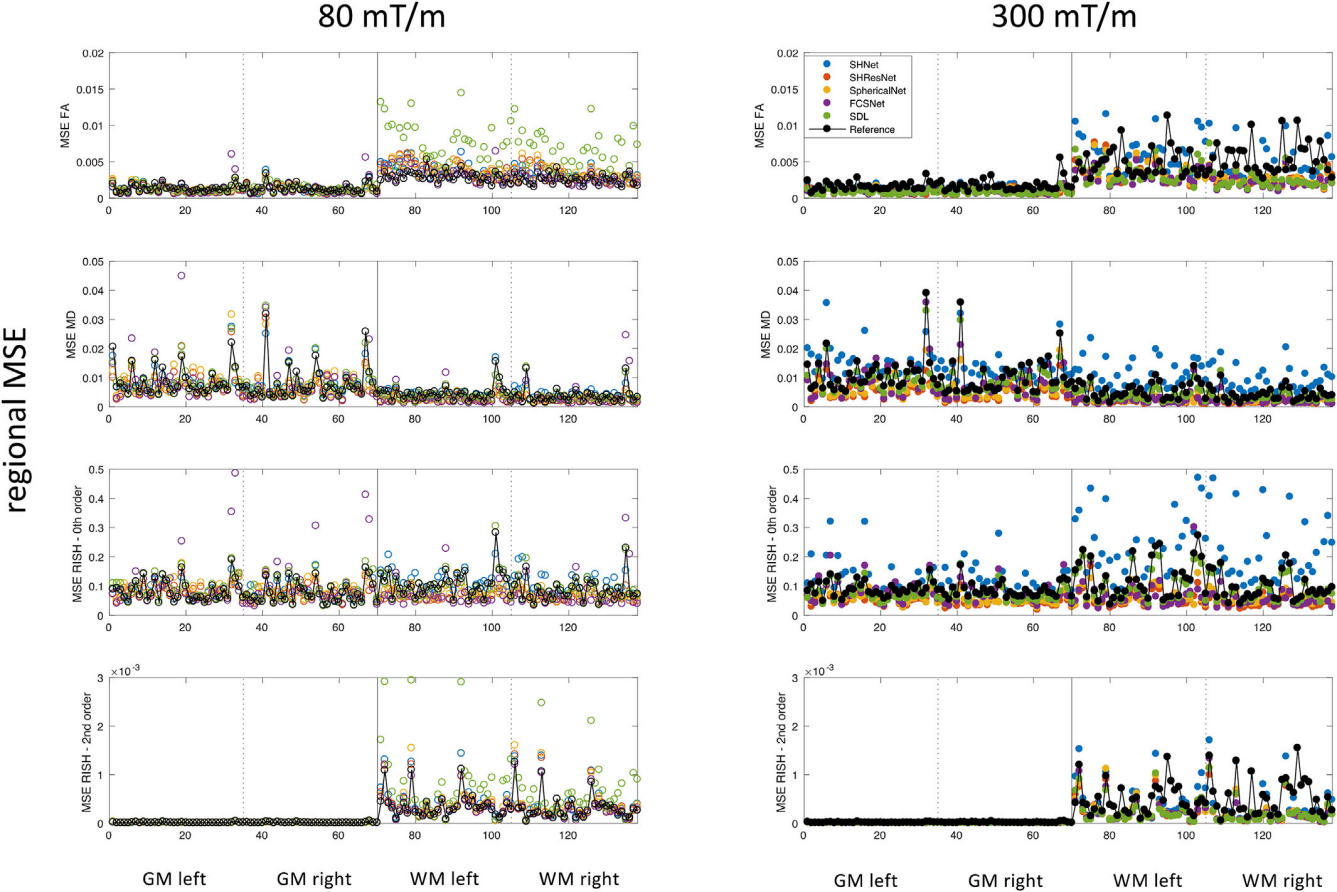
Results of the scanner-to-scanner mapping: Regional MSE for FA, MD, R0, and R2 (rows); median MSE across subjects per ROI per algorithm; for predictions of the 80 mT/m data (left, open circles) and the 300 mT/m data (right, closed circles). The different methods are represented with different colours SHNet (blue), SHResNet (red), SphericalNet (orange), FCSNet (purple), SDL (green), reference (black). Regions 1 to 35 and 36 to 71 are left and right GM regions respectively, regions 72 to 107 and 108 to 143 are left and right WM regions respectively (see Supplementary Table 3).

#### 3.1.3 Local evaluation

Distributions of the localised MNE and MSE were computed across subjects per evaluated algorithm, Fig. 8 shows the median and width (95^th^ percentile) of the MNE (top) and MSE (bottom) for 80 mT/m and 300 mT/m. This analysis was restricted to the white matter, and all ROIs that scored systematically poor (cf. Fig. 7) were excluded. Table 6 summarises the results.

**Fig. 8:**
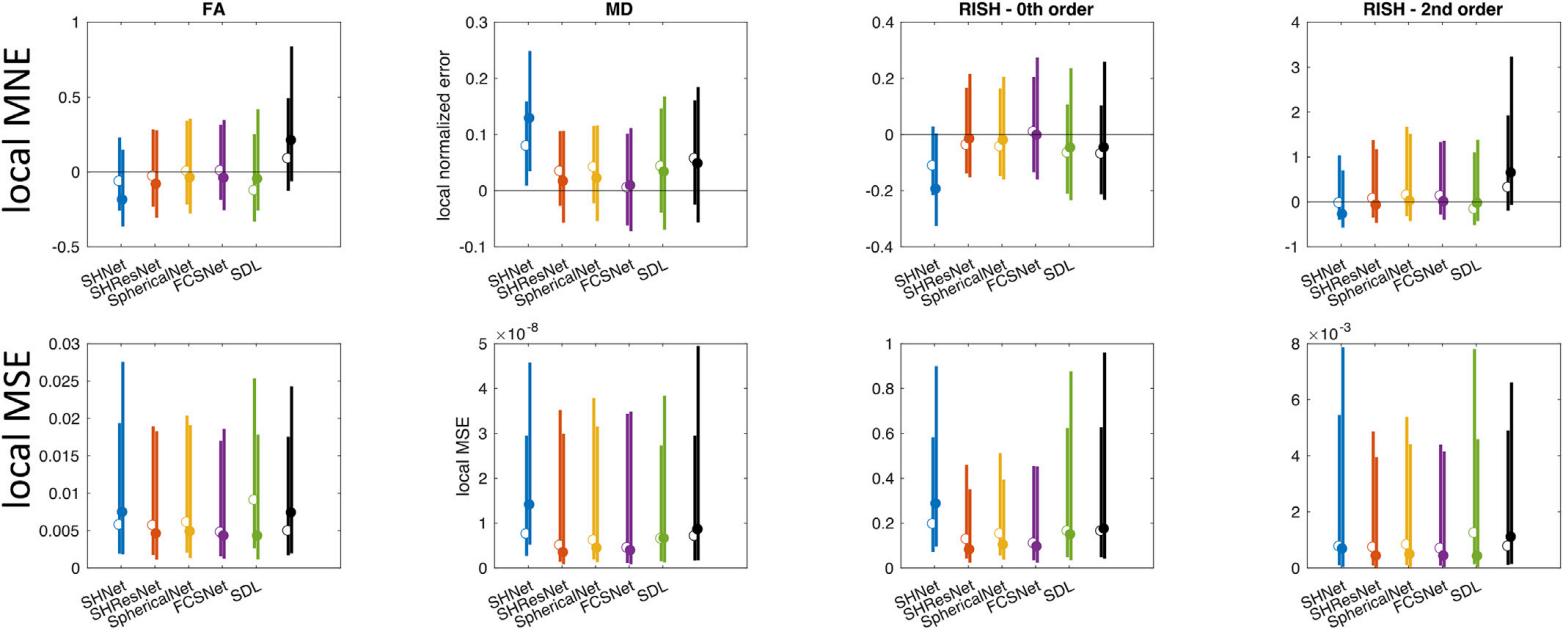
Results of the scanner-to-scanner mapping: Local MSE and MNE for FA, MD, R0, and R2 (columns), across WM (excluding problematic ROIs) and all subjects. The different methods are represented with different colours: SHNet (blue), SHResNet (red), SphericalNet (orange), FCSNet (purple), SDL (green), and reference (black). (a) Local MNE distributions for the 80 mT/m predictions (top) and 300 mT/m predictions (bottom). (b) The bars show the 95^th^ percentile of the MNE and MSE distributions for the 80 mT/m predictions (median indicated by open circles) and the 300 mT/m predictions (median indicated by closed circles).

**Table 6:**
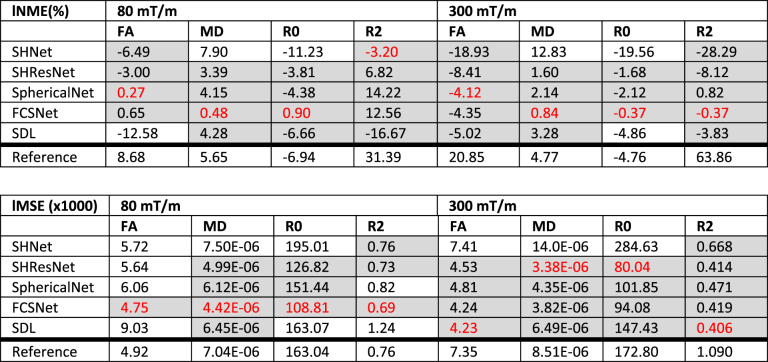
Results of the scanner-to-scanner mapping: median localised MNE and median localised MSE for each algorithm and metric, across subjects. Algorithms performing better than the reference (trilinear and SH interpolation) are highlighted in gray, the best performing algorithm for each metric is highlighted in red.

The harmonisation algorithms generally have a median localised MNE closer to zero compared to the reference, and thus perform better than simple interpolation (Fig. 8, top). For each metric, at least one algorithm has a median localised MNE lower than 5%, with minimal errors less than 1% (Table 6). However, localised MNE of more than 15% are observed for all algorithms and evaluated metrics. The performance of the different harmonisation techniques varies widely (up to an order of magnitude) across the metrics and scanners, without one technique consistently outperforming all others. Indeed, 3 out of the 5 algorithms achieve the lowest median localised MNE error in at least one of the metrics. SHResNet, SphericalNet and FCSNet outperformed the reference for all metrics and both scanners, where FCSNet is the most consistent well-performing algorithm. Predictions for the 300 mT/m system are wider than for the 80 mT/m system (compare different bars in Fig. 8 top).

Fig. 8 bottom shows distributions of localised MSE evaluating both accuracy and precision, and most algorithms outperform the reference for both scanners. For 80 mT/m predictions, FCSNet outperformed all algorithms; for 300 mT/m predictions, SHResNet and SDL both outperformed the others in 2 of the 4 metrics.

### 3.2 Spatial- and angular resolution enhancement

#### 3.2.1 Local evaluation

**Fig. 9:**
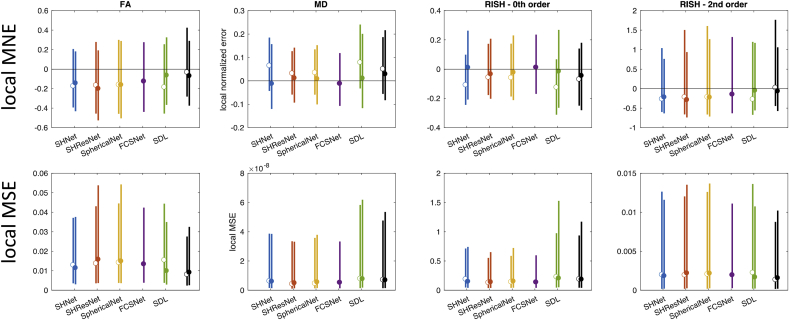
Results of the spatial and angular resolution enhancement: Local MSE and MNE for FA, MD, R0, and R2 (columns), across WM (excluding problematic ROIs) and all subjects. The different methods are represented with different colours: SHNet (blue), SHResNet (red), SphericalNet (orange), FCSNet (purple), SDL (green), and reference (black). The bars show the 95^th^ percentile of the MNE and MSE distributions for the 80 mT/m predictions (median indicated by open circles) and the 300 mT/m predictions (median indicated by closed circles).

Fig. 9 shows the median and width of the localised MNE and MSE distributions, and the medians are also reported in Table 7. The performance for this task is poorer than the scanner-to-scanner mapping with errors being larger. The harmonisation algorithms do not always outperform the reference. For the 80 mT/m prediction, the reference interpolation even outperforms the harmonisation approaches for two out of four diffusion metrics, specifically the metrics related to anisotropy. For metrics MD and R0, SHResNet and SphericalNet consistently outperform the reference for both scanners. Overall, the 300 mT/m predictions have higher accuracy with a maximal median localised MNE of 7%. Again, a wide variability of all methods across the different metrics and scanners could be observed without one method outperforming the others.

**Table 7:**
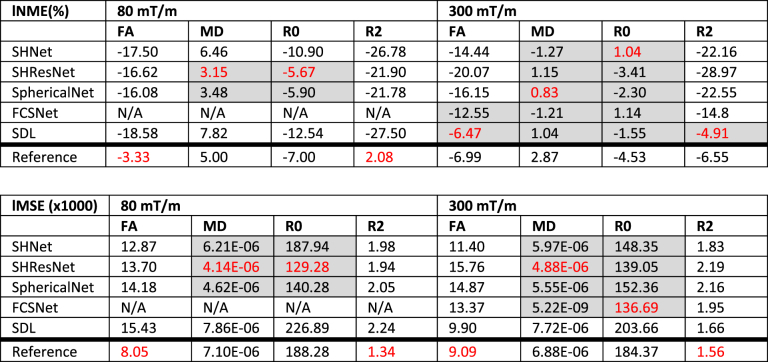
Results of the spatial and angular resolution enhancement: median localised MNE and median localised MSE for each algorithm and metric, across subjects. Algorithms performing better than the reference are highlighted in gray, the best performing algorithm for each metric is highlighted in red.

## 4 Discussion

With the increasing prevalence of MRI research systems capable of collecting diffusion MRI data, comes the potential of combining data sets of much larger size than could ever be collected at one centre alone. Several studies have reported a variability between diffusion measurements acquired at different scanners and sites (Chen et al., 2014; Mirzaalian et al., 2017, 2016; Vollmar et al., 2010), even with comparable protocols. When protocols differ substantially because of updated hardware and software, more ‘historical’ data can potentially be enhanced by learning features from ‘state-of-the-art’ data (Alexander et al., 2017).

As a result, the interest in developing methods to establish a mapping between different scanners and protocols is continuously growing (Jahanshad et al., 2013; Kochunov et al., 2014; Venkatraman et al., 2015; Jenkins et al., 2016; Pohl et al., 2016; Fortin et al., 2017; Mirzaalian et al., 2016). Harmonisation on groups of different subjects scanned on different scanners can be performed by finding spatial correspondence between subjects by registration to an atlas, and relies on the assumption that the diffusion measurements between matched groups (in age, gender, etc.) are statistically different only due to scanner-differences. In a group of travelling subjects as presented here, the confounding factor of inter-subject differences is removed and spatial correspondence can be obtained more directly. While a travelling control group does not preclude group-level harmonisation strategies, it should allow scanner-specific effects to be captured with fewer subjects. In this work, we have presented a benchmarking database of acquisitions of the same healthy controls scanned on different scanners with different maximum gradient strengths and protocols.

### 4.1 Data

Scanning a ‘travelling head’ on different scanners should ideally be performed in quick succession to avoid intermediate software updates and age-related effects. In this work, the average time between acquisitions on scanners a) and b), and a) and c) was 21 and 22 months, respectively. Whereas the time between scans b) and c) was very short, the time difference with scan a) was longer. Age-related changes during this period might be present but are assumed to be small compared to the source of variance introduced by cross-scanner and cross-protocol differences. By including adult subjects with an average age of 25.7 years we have strived to minimise such confounds; previous studies have shown that age-related FA and MD changes in several white matter structures reach a plateau around the age of 25 (Lebel et al., 2008). Similarly, variabilities that might occur when scanning subjects at different time points during a day (Thomas et al., 2018) are assumed to be small compared to cross-scanner variability in this study. None of the scanners had software upgrades during the course of the study.

Scan-rescan experiments in quick succession can provide information on the inherent measurement variability of a particular scanner and sequence, and as such give an estimate of the lower bound of harmonisation performance. We performed a preliminary analysis on rescans of the b = 1200 s/mm^2^ data for 3 subjects on the 300 mT/m system, of which one subject overlapped with the evaluated subjects. The global MSE (Fig. 6, bottom) shows that the best performing techniques approach the range of scan-rescan reproducibilities for all metrics, with an overall lower performance in terms of FA. Median localised MNE (not shown) varied from 2 to 5% for FA in agreement with the literature (Kochunov et al., 2014). We have adopted the same normalization procedure of registering the data to the 80 mT/m space (both the scan- and rescan). Better overlap of inter-scanner data could be achieved than for intra-scanner data, which might have partially contributed to lower median MSE values.

In addition to travelling heads, physical phantoms can be used to detect scanner-specific variabilities and changes, and to correct for such variabilities with harmonisation approaches. While such phantoms do not suffer from age-related or time-of-day effects, they are incapable of fully capturing the complexity of biological tissue and regional differences associated with this complexity. Furthermore, it can be non-trivial to translate the differences observed in physical phantoms to in-vivo acquisitions. Nevertheless, learning a scanner-to-scanner mapping from a travelling phantom would be an interesting alternative challenge, and ideally both in-vivo travelling head- and physical phantom acquisitions could be combined to assess variabilities and evaluate harmonisation approaches.

The acquisition parameters of the matching resolution cross-scanner (ST) protocols were harmonised as closely as possible in terms of b-value, TE, TR, spatial resolution, and angular resolution. However, slight variations between the ST protocols on different scanners and vendors remained. While this could introduce additional variability in the measurements, it also mimics common more subtle variabilities between scanner sites. This allows us to test whether harmonisation approaches are robust to such changes.

While the database was here used as a testbed for harmonisation algorithms, it could contribute to answering alternative questions as well. Recently, part of the database has been utilised to investigate the dependency of Meyer's loop tractography on imaging protocol and hardware (Chamberland et al., 2018).

### 4.2 Preprocessing

Minimally preprocessed data were made available to the entrants of the challenge, but the raw (unprocessed) data will be made available upon request. The data preprocessing pipeline can have an effect on the degree to which the datasets are comparable prior to data harmonisation (Jenkins et al., 2016), as differences in preprocessing can induce differences in data across acquisitions that are hard to harmonise *a posteriori*. For example, different methods were used to correct for susceptibility distortions between the 40 mT/m system and the other systems, because a reversed phase encoding b0 image was not available for the former. In the current study, we did not find that regions affected by susceptibility distortions (e.g. frontal regions) performed systematically poorer than other regions, but such differences will likely have an effect when performing multi-centre studies. Investigating this effect is subject to future work.

While the preprocessing pipeline included the most commonly performed steps such as motion correction and eddy current- and susceptibility distortion correction, the importance of correcting for other artifacts has been stressed in various works; e.g. Gibbs ringing (Kellner et al., 2016; Perrone et al., 2015; Veraart et al., 2016a), signal drift (Vos et al., 2016), and others (Andersson, 2014; Le Bihan et al., 2006; Pierpaoli, 2010; Tax et al., 2016). Manual inspection of the data did not reveal any gross artifacts such as slice intensity dropouts, but artifacts could have a less visible impact on signal intensities and as such additional preprocessing steps could be included in the preprocessing pipeline.

The use of different software tools for the different preprocessing steps resulted in multiple re-samplings and interpolations of the data. Ideally, the different warps (of motion/eddy current distortion correction, gradient nonlinearity distortion correction, and registration to a common space) should be concatenated and performed within a single interpolation step. However, to the best of our knowledge, there is currently no consensus on the order of performing motion/eddy current distortion correction and gradient nonlinearity distortion correction (Rudrapatna et al., 2018), and the ‘best’ practice likely depends on the amount of subject motion.

Gradient nonlinearity in the 300 mT/m system not only causes geometrical image distortions, but also spatially varying b-vectors and b-values (Bammer et al., 2003). To simplify the tasks, the variance in diffusion weighting was not taken into account in the current comparison, and might be a possible explanation for the greater variance in the 300 mT/m predictions. This information could potentially improve harmonisation with the 300 mT/m system and will be made available.

For the registration to a common space, we have qualitatively compared different toolboxes, degrees of freedom (linear vs affine), and input images (FA, B0, mean of the DWI), and observed that the quality of registration varied with the input image and degrees of freedom. Here, we used the mean of the DWI images, but other choices are possible, such as b0 images, FA, or a multi-contrast approach. Rigid (translation and rotation) registration to a common space for each subject was generally not sufficient to achieve good alignment. This indicates that residual distortions remain that were not corrected during preprocessing. Nonlinear registration was used for the 40 mT/m data to simultaneously correct for susceptibility distortions in the phase encoding direction, while affine registration was used for all the other data. Full nonlinear registration is envisioned to give better overlap, but the interaction between local deformations and the orientational information present in the DWIs adds a layer of complexity. Therefore, for this work, we decided to stick to affine transformations, but addressing the remaining distortions between scanners after preprocessing is an important issue general to harmonisation that should be addressed in future work. In Supplementary Material 4, we show a qualitative comparison of the registrations for one subject, and we report the decrease of mean squared error that could be achieved with full nonlinear registration of the test subjects, compared to the registration performed in the current evaluation. The MSE were all lower than 0.016, and the 300 mT/m ST registration showed the lowest improvement in MSE with full nonlinear registration, followed by 80 mT/m SA, 300 mT/m SA, and 40 mT/m ST. We also report the mean of the Jacobian determinant, reflecting how much local deformation was necessary.

### 4.3 Evaluation procedure

The evaluation in this study was performed on a global, regional, and local level, and different diffusion metrics were derived. The results suggest that the relative performance of algorithms strongly depends on the metric evaluated, and that the harmonisation can thus be tuned towards the metric of interest. The current work focused on the presentation of the harmonisation-benchmark database and a first evaluation of harmonisation algorithms on this database, where the evaluation was specifically targeted at metrics that are most widely used in clinical studies (e.g. DTI features from single-shell data). This can be extended in future work in multiple ways. Evaluation of higher-order metrics, e.g. RISH metrics derived from the 4^th^ and 6^th^ spherical harmonic order, could provide further insight into the performance of the signal harmonisation, but the precision of their estimates can be lower. For higher order metrics, it would be interesting to investigate the impact of the number of gradient directions when the resolution is higher and the SNR lower, as is the case in the resolution enhancement task. In addition, as multi-shell dMRI data is becoming more readily available, harmonisation algorithms should be extended to accommodate data acquired with multiple b-values. The presented database includes dMRI data with multiple b-values, and therefore allows an evaluation of such algorithms in future work. Finally, the diffusion signals could be compared directly. However, the actual diffusion measurements include B1 transmit-, amplifier-, and receive effects, and their multiplicative scaling is arbitrary. An additional prediction of this scale for each scan would be necessary and therefore adds another layer of complexity. In this first evaluation we have therefore opted for the evaluation of summary metrics that are independent of this scale, but the evaluation of individual predicted diffusion signals can be performed in future work.

In this work, the aim was to harmonise the DWIs directly in native space (as for example also done in Mirzaalian et al. (2016)), as opposed to harmonising feature maps such as DTI-derived metrics (Fortin et al., 2017; Jahanshad et al., 2013; Kochunov et al., 2014; Pohl et al., 2016; Venkatraman et al., 2015). The direct harmonisation of DWIs is beneficial in that any metric can later be compared between groups, and it potentially allows to better capture certain complex variations as opposed to harmonising particular features alone. Performing the mapping in atlas space can be beneficial if the data is analysed in this common space to avoid the additional back-projection step to native space (e.g. in voxel-based analysis), but harmonisation in native space allows the use of approaches that analyse the data without registration to an atlas, e.g. tract-based approaches where tractography is performed and mean-tract values are compared across subjects. In this work, we evaluate predictions of the data at the reference site and compare to the acquired ground truth in native space. Evaluating algorithms by their ability to remove statistical group differences would be possible, but would likely benefit from larger cohorts. The challenge setup is therefore in part motivated by the sample size.

MNE and MSE were computed to assess accuracy and precision. In addition to comparing low-level error metrics and reduce this down to a score for the overall performance of a harmonisation approach, an alternative evaluation can be targeted to a particular task; for example, the harmonisation of values along selected tracts reconstructed with tractography or the ability to discriminate patients and controls. While this would give a clearer picture of harmonisation performance for the task at hand, it remains unclear how these results extend among different tasks.

Misalignments between scanners both in the training data and test data can influence the results. For this reason, a registration step was included in the preprocessing pipeline so that misregistration would affect all harmonisation algorithms to the same extent. While misalignments can vary in a non-systematic way owing to inter-individual differences, systematically bad performing regions may have suffered from this to a larger extent. None of the entrants reported additional registration steps, so it can be expected that misregistration would affect all harmonisation algorithms to a similar degree. It is envisioned that optimised registration will not only improve the learned mapping between scanners, but may also reduce the errors observed in the evaluation stage.

### 4.4 Implications and recommendations

From the results, some broad trends become apparent. For the scanner-to-scanner mapping, the best performing algorithms give a good consistency across subjects (in terms of global MSE, Fig. 6), but less consistency across regions (Fig. 7). Fig. 7 shows differences in regional error scores for all algorithms between WM and GM: metrics that describe anisotropy (i.e. FA and R2) have larger MSE in WM, whereas MD and R0 have more comparable MSE in WM vs GM. For most metrics and harmonisation algorithms, median localised MNE of <5% could be obtained for both scanners (with values of <1% for the best performing algorithms), however, also localised MNE of >15% were observed.

For the angular- and spatial-resolution enhancement, median localised MNE of <5% and <1% could be obtained for the 80 mT/m and 300 mT/m scanner respectively, but only for isotropic measures (MD and R0). A possible explanation is that local variations in MD and R0 are smaller (e.g. at WM/GM interfaces) such that spatial misalignment problems could be less pronounced. Machine learning techniques probably outperform linear interpolation because they are able to correct global offsets. In contrast, FA and R2 are anisotropy measures that have clear ‘edges’ at WM/GM interfaces, hence co-registration issues can be amplified. FCSNet is most consistently well-performing, and its use of a larger local neighbourhood could be beneficial to ameliorate such issues. The good performance of the reference interpolation compared to machine learning-based methods might indicate that some blurring might improve the results, but too much blurring is detrimental in most cases (Supplementary Fig. 1).

It is important to consider these numbers in the light of the magnitudes of the effects-of-interest in group studies, which are often smaller than the 15% mentioned above. While harmonisation approaches should remove differences across sites, inter-individual differences and differences between the healthy control and disease population should be preserved after harmonisation. We see our database as a first significant step towards a general benchmarking of harmonisation methods in healthy controls. A breadth of different microstructural architectural paradigms can already be seen across the young healthy brain; from isotropic CSF, to isotropic gray matter, to white matter with multiple crossing fibres, to white matter with single fibres and different degrees of anisotropy, as well as different degrees of partial voluming with isotropic configurations. Depending on the degree of microstructural changes, this may or may not fall within the range of microstructural architectural paradigms that can be predicted from healthy controls. Nevertheless, it would be reasonable to expect that if harmonisation algorithms have been reported to work well for the dataset present here, that they will likely work well on training databases that include a wider age range and pathological cases. This could also potentially be assessed by synthetic experiments and data augmentation, as e.g. in Mirzaalian et al. (2016).

To assess the impact on real-world analysis tasks, such as group- or longitudinal studies where large samples are necessary to detect small effects, power analyses could be performed. For example, one could work out the reduction in the total number of participants needed across the scanners to see a given effect size. Alternatively, for a fixed number of participants, one could compute what effect size could be detected. The exact computation depends on the choice of effect size (Lakens, 2013). Scan-rescan errors can serve as a good estimate of the inherent variability and lower bound performance of all harmonisation algorithms, and therefore of the smallest changes that can realistically be observed. The current results suggest that in scanner-to-scanner mapping, harmonisation approaches can reduce the variability for the metrics investigated, with the best performing algorithms approaching the range of scan-rescan reproducibilities.

Based on the exercise of data harmonisation as performed in this study, we highlight a few recommendations for data harmonisation in future multi-centre studies, covering different stages of the analysis pipeline:

1) Variability between acquisitions on different scanners should be reduced by matching of protocols as much as possible. This can be a challenge in itself as different platforms do not always allow perfect matching. One should consider main parameters such as spatial resolution, TE, number and distribution of gradient directions, and b-value; but also other parameters such as parallel imaging, multiband, partial Fourier, and reconstruction settings. It is envisioned that better matching will lead to better *a priori* harmonisation, but a reduction in measurement variability can still be achieved in the case of non-perfect matching, as illustrated in this study. The question which parameters have the largest effect is challenging to answer and can be the subject of future studies.
2) Because of the effect sizes typically found in diffusion MRI studies, a travelling head and/or travelling phantom study should ideally be performed on all the scanners involved in the study, as well as a characterisation of scan-rescan variability. This requires subjects to be scanned and re-scanned across different sites and between protocols, but potentially also before and after every software update. Training datasets on the order of 10 subjects, as used in this work, could be a logistic and financial burden on a study if the sample size is small. However, it could be argued that if a research question requires a seriously large sample size that demands a multi-site study, the proportional cost and administrative burden of scanning a few subjects on both sites is manageable and could be justified by the advantage of reliable harmonisation and increased statistical power. Unless the patient group is exceedingly rare and a multi-site study is required to achieve even a small sample size, studies with small sample sizes may as well be performed at a single site. Future work should assess this trade-off, and compare to a group-based approach where different subjects scanned on different scanners are matched and used for harmonisation.
3) Accurate characterisation and detection of artifacts on each scanner is important to improve *a priori* harmonisation (that is, prior to applying harmonisation algorithms as described in this study). To this end, acquiring additional data for improved artefact correction is recommended. One can think of acquiring reversed-phase encoding images or field maps for the correction of susceptibility distortions (Andersson et al., 2003; Irfanoglu et al., 2015), noise maps for the characterisation of noise distributions (Froeling et al., 2016) and denoising (e.g. (St-Jean et al., 2016; Veraart et al., 2016c)), and additional gradient directions to improve outlier detection and reduce the effect of outlier rejection (Chang et al., 2005; Collier et al., 2015; Mangin et al., 2002; Sairanen et al., 2018; Tax et al., 2015). Not appropriately correcting for such artifacts can result in increased variability and can impose extra challenges to harmonisation algorithms.
4) Likewise, differences in preprocessing and model estimation strategies between different scanners can introduce additional variability, and such pipelines should be matched whenever possible.
5) Point-to-point mapping between different subjects and different scanners by means of a registration procedure deserves attention; residual distortions after preprocessing can be observed.
6) In the case of a clear hypothesis of the region or tract involved in the phenomenon under investigation one could perform a more in-depth investigation of measurement variability than global error measures.

### 4.5 Obtaining the data

The database is available to the public, to encourage further development of harmonisation approaches and to further evaluate e.g. the use of machine learning vs alternative methods, using spatial context vs not using spatial context, and employing data augmentation techniques. Information on how to obtain the data can be found on the following webpage: https://www.cardiff.ac.uk/cardiff-university-brain-research-imaging-centre/research/projects/cross-scanner-and-cross-protocol-diffusion-MRI-data-harmonisation.

## 5 Conclusion

In conclusion, cross-scanner and cross-protocol measurement variability challenges multi-centre studies. In this study, we have presented a benchmarking database to test and evaluate harmonisation approaches for cross-scanner and cross-protocol mapping. The harmonisation approaches proposed significantly reduce variability in multi-vendor diffusion scans with comparable protocols, but challenges in spatial- and angular resolution enhancement of features that characterise anisotropy remain. Before widespread deployment of harmonisation schemes for large multi-site studies, it would be important to perform more rigorous testing, e.g. in other training sets. In future work, the benchmarking database will be utilised to evaluate harmonisation approaches for multi-shell diffusion MRI data and the influence of preprocessing on the performance of harmonisation.

## Figures and Tables

**figs1:**
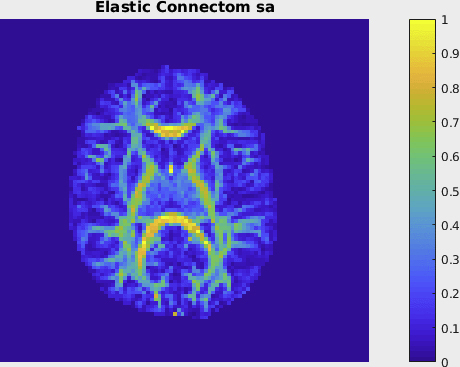


**figs2:**
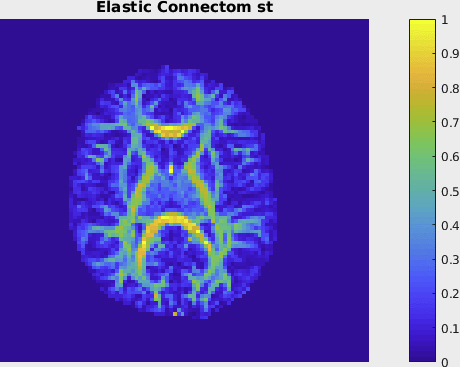


**figs3:**
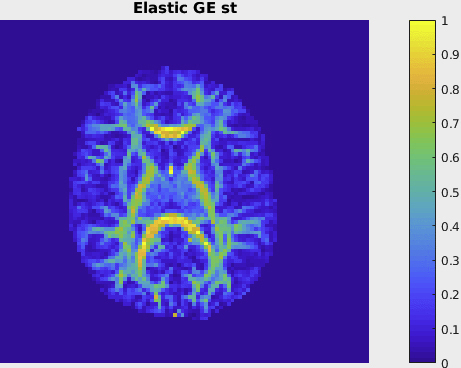


**figs4:**
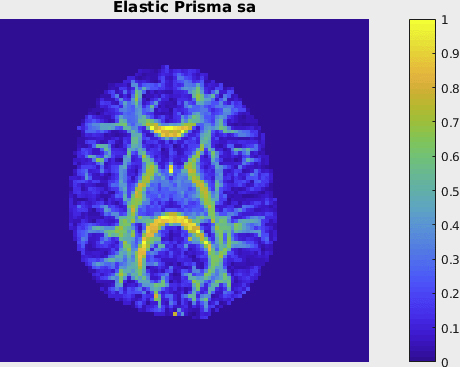


**figs5:**
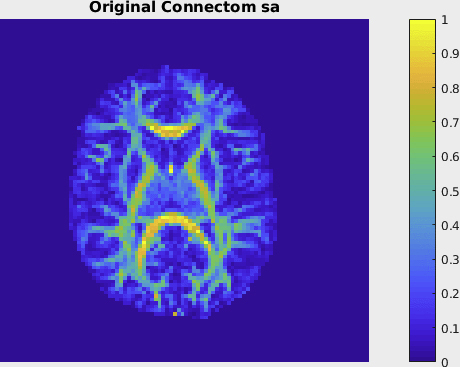


**figs6:**
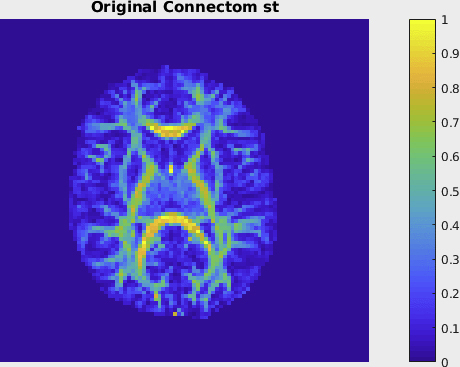


**figs7:**
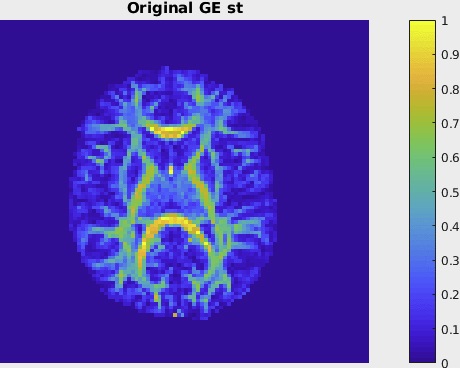


**figs8:**
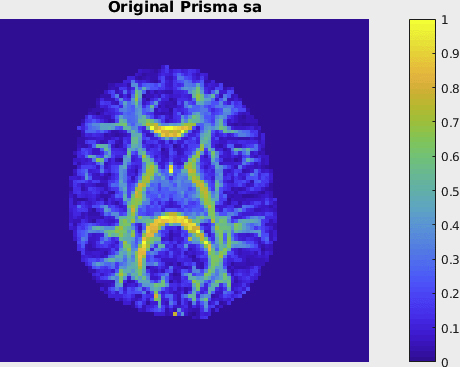


## Footnotes

1 Here we use the word ‘harmonisation’ in the context of finding a mapping between datasets, irrespective of their difference in quality.

2 These data were collected for the 'Diffusion MRI Harmonisation Challenge', an initiative presented at the 2017 Computational Diffusion MRI Workshop of the MICCAI conference in Quebec City, Canada https://projects.iq.harvard.edu/cdmri2017/home.
