# Supplementary material for: Cross-scanner and cross-protocol diffusion MRI data harmonisation: A benchmark database and evaluation of algorithms
Source: Neuroimage. 2019 Jul 15;195:285–99. doi: 10.1016/j.neuroimage.2019.01.077 (PMC6556555; doi:10.1016/j.neuroimage.2019.01.077)
Supplement: Multimedia component 1 [file mmc1.docx]

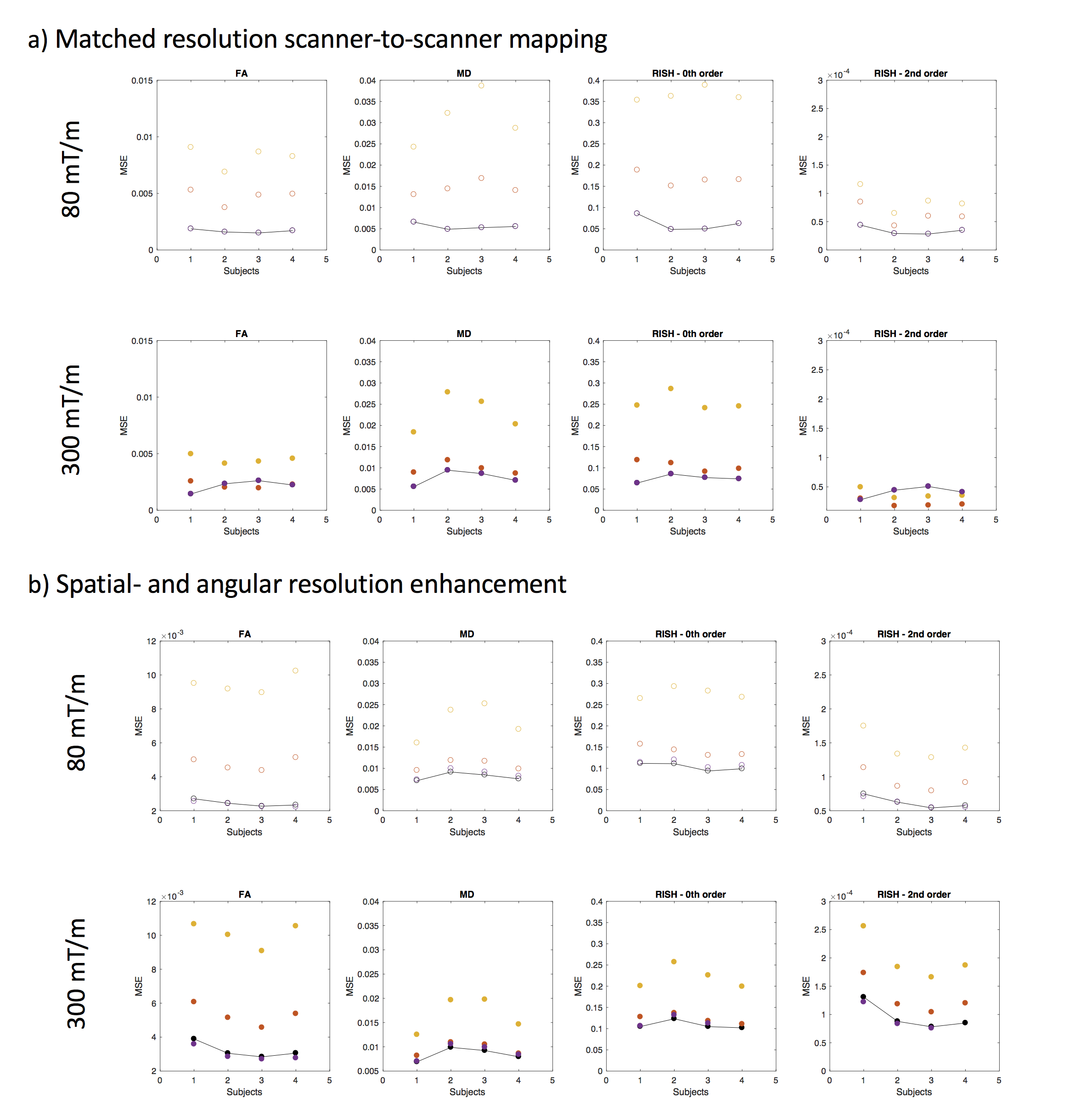


Supplementary Figure 1: Influence of blurring and interpolation on the global evaluation results for task 1 (a) and task 2 (b). Black: reference as used in the manuscript, trilinear interpolation. Purple: reference with sinc interpolation gives very similar results. Orange: reference blurred with a Gaussian filter with sigma = 1. Yellow: reference blurred with a Gaussian filter with sigma = 2.

| SNR estimates on the b0 image (median) | | | | | |
| --- | --- | --- | --- | --- | --- |
|  | GE 40 mT/m | Siemens 80 mT/m | | Siemens 300 mT/m | |
| **Subject** | **Standard (ST)** | **Standard (ST)** | **State-of-the-art (SA)** | **Standard (ST)** | **State-of-the-art (SA)** |
| A | 47.7967 | 41.9524 | 19.6226 | 47.8768 | 16.4541 |
| B | 43.6129 | 43.0045 | 22.9458 | 42.3547 | 16.1561 |
| C | 51.5113 | 47.3700 | 23.3669 | 44.2372 | 15.5905 |
| D | 48.8227 | 41.7349 | 21.6140 | 46.5823 | 16.4803 |
| E | 54.9576 | 47.5598 | 22.9148 | 45.6365 | 15.8360 |
| F | 50.2696 | 40.9820 | 20.4252 | 43.2030 | 16.2903 |
| G | 58.5557 | 41.4180 | 20.1591 | 45.1596 | 17.3791 |
| H | 45.4300 | 45.0503 | 23.1948 | 45.5411 | 16.3516 |
| I | 48.3117 | 42.4160 | 21.1734 | 44.0117 | 15.6669 |
| J | 49.7617 | 46.6988 | 23.2739 | 46.1664 | 17.3340 |
| K | 46.4187 | 40.9219 | 20.9008 | 41.6043 | 15.1554 |
| L | 43.0682 | 42.4386 | 21.7728 | 41.2593 | 15.0620 |
| M | 46.7704 | 43.4654 | 21.7877 | 46.8499 | 17.2817 |
| N | 45.6787 | 44.8192 | 23.6014 | 44.0857 | 15.8426 |

Supplementary Table 1: B0 signal divided by the noise standard deviation estimation on the non-preprocessed data, median within brain mask.

| Number of voxels in brain masks generated from the GE T1, excluding the cerebellum | | | | | |
| --- | --- | --- | --- | --- | --- |
|  | GE 40 mT/m | Siemens 80 mT/m | | Siemens 300 mT/m | |
| **Subject** | **Standard (ST)** | **Standard (ST)** | **State-of-the-art (SA)** | **Standard (ST)** | **State-of-the-art (SA)** |
| A | 83470 | 83470 | 341469 | 83470 | 667661 |
| B | 94893 | 94893 | 388498 | 94893 | 758983 |
| C | 87912 | 87912 | 359933 | 87912 | 703116 |
| D | 84279 | 84279 | 345168 | 84279 | 674047 |
| E | 88744 | 88744 | 363666 | 88744 | 709773 |
| F | 87354 | 87354 | 357507 | 87354 | 698629 |
| G | 99959 | 99959 | 408962 | 99959 | 799455 |
| H | 92466 | 92466 | 378561 | 92466 | 739467 |
| I | 94097 | 94097 | 385322 | 94097 | 752609 |
| J | 92574 | 92574 | 379246 | 92574 | 740375 |
| K | 89928 | 89928 | 368190 | 89928 | 719303 |
| L | 87260 | 87260 | 357106 | 87260 | 697893 |
| M | 98753 | 98753 | 404647 | 98753 | 789851 |
| N | 85249 | 85249 | 349149 | 85249 | 681834 |

Supplementary Table 2: Number of voxels within brain mask

| ROI nr | Freesurfer Region |
| --- | --- |
|  | 1001 ctx-lh-bankssts |
|  | 1002 ctx-lh-caudalanteriorcingulate |
|  | 1003 ctx-lh-caudalmiddlefrontal |
|  | 1004 ctx-lh-corpuscallosum |
|  | 1005 ctx-lh-cuneus |
|  | 1006 ctx-lh-entorhinal |
|  | 1007 ctx-lh-fusiform |
|  | 1008 ctx-lh-inferiorparietal |
|  | 1009 ctx-lh-inferiortemporal |
|  | 1010 ctx-lh-isthmuscingulate |
|  | 1011 ctx-lh-lateraloccipital |
|  | 1012 ctx-lh-lateralorbitofrontal |
|  | 1013 ctx-lh-lingual |
|  | 1014 ctx-lh-medialorbitofrontal |
|  | 1015 ctx-lh-middletemporal |
|  | 1016 ctx-lh-parahippocampal |
|  | 1017 ctx-lh-paracentral |
|  | 1018 ctx-lh-parsopercularis |
|  | 1019 ctx-lh-parsorbitalis |
|  | 1020 ctx-lh-parstriangularis |
|  | 1021 ctx-lh-pericalcarine |
|  | 1022 ctx-lh-postcentral |
|  | 1023 ctx-lh-posteriorcingulate |
|  | 1024 ctx-lh-precentral |
|  | 1025 ctx-lh-precuneus |
|  | 1026 ctx-lh-rostralanteriorcingulate |
|  | 1027 ctx-lh-rostralmiddlefrontal |
|  | 1028 ctx-lh-superiorfrontal |
|  | 1029 ctx-lh-superiorparietal |
|  | 1030 ctx-lh-superiortemporal |
|  | 1031 ctx-lh-supramarginal |
|  | 1032 ctx-lh-frontalpole |
|  | 1033 ctx-lh-temporalpole |
|  | 1034 ctx-lh-transversetemporal |
|  | 1035 ctx-lh-insula |
|  |  |
|  | 2000 ctx-rh-unknown |
|  | 2001 ctx-rh-bankssts |
|  | 2002 ctx-rh-caudalanteriorcingulate |
|  | 2003 ctx-rh-caudalmiddlefrontal |
|  | 2004 ctx-rh-corpuscallosum |
|  | 2005 ctx-rh-cuneus |
|  | 2006 ctx-rh-entorhinal |
|  | 2007 ctx-rh-fusiform |
|  | 2008 ctx-rh-inferiorparietal |
|  | 2009 ctx-rh-inferiortemporal |
|  | 2010 ctx-rh-isthmuscingulate |
|  | 2011 ctx-rh-lateraloccipital |
|  | 2012 ctx-rh-lateralorbitofrontal |
|  | 2013 ctx-rh-lingual |
|  | 2014 ctx-rh-medialorbitofrontal |
|  | 2015 ctx-rh-middletemporal |
|  | 2016 ctx-rh-parahippocampal |
|  | 2017 ctx-rh-paracentral |
|  | 2018 ctx-rh-parsopercularis |
|  | 2019 ctx-rh-parsorbitalis |
|  | 2020 ctx-rh-parstriangularis |
|  | 2021 ctx-rh-pericalcarine |
|  | 2022 ctx-rh-postcentral |
|  | 2023 ctx-rh-posteriorcingulate |
|  | 2024 ctx-rh-precentral |
|  | 2025 ctx-rh-precuneus |
|  | 2026 ctx-rh-rostralanteriorcingulate |
|  | 2027 ctx-rh-rostralmiddlefrontal |
|  | 2028 ctx-rh-superiorfrontal |
|  | 2029 ctx-rh-superiorparietal |
|  | 2030 ctx-rh-superiortemporal |
|  | 2031 ctx-rh-supramarginal |
|  | 2032 ctx-rh-frontalpole |
|  | 2033 ctx-rh-temporalpole |
|  | 2034 ctx-rh-transversetemporal |
|  | 2035 ctx-rh-insula |
|  |  |
|  | 3000 wm-lh-unknown |
|  | 3001 wm-lh-bankssts |
|  | 3002 wm-lh-caudalanteriorcingulate |
|  | 3003 wm-lh-caudalmiddlefrontal |
|  | 3004 wm-lh-corpuscallosum |
|  | 3005 wm-lh-cuneus |
|  | 3006 wm-lh-entorhinal |
|  | 3007 wm-lh-fusiform |
|  | 3008 wm-lh-inferiorparietal |
|  | 3009 wm-lh-inferiortemporal |
|  | 3010 wm-lh-isthmuscingulate |
|  | 3011 wm-lh-lateraloccipital |
|  | 3012 wm-lh-lateralorbitofrontal |
|  | 3013 wm-lh-lingual |
|  | 3014 wm-lh-medialorbitofrontal |
|  | 3015 wm-lh-middletemporal |
|  | 3016 wm-lh-parahippocampal |
|  | 3017 wm-lh-paracentral |
|  | 3018 wm-lh-parsopercularis |
|  | 3019 wm-lh-parsorbitalis |
|  | 3020 wm-lh-parstriangularis |
|  | 3021 wm-lh-pericalcarine |
|  | 3022 wm-lh-postcentral |
|  | 3023 wm-lh-posteriorcingulate |
|  | 3024 wm-lh-precentral |
|  | 3025 wm-lh-precuneus |
|  | 3026 wm-lh-rostralanteriorcingulate |
|  | 3027 wm-lh-rostralmiddlefrontal |
|  | 3028 wm-lh-superiorfrontal |
|  | 3029 wm-lh-superiorparietal |
|  | 3030 wm-lh-superiortemporal |
|  | 3031 wm-lh-supramarginal |
|  | 3032 wm-lh-frontalpole |
|  | 3033 wm-lh-temporalpole |
|  | 3034 wm-lh-transversetemporal |
|  | 3035 wm-lh-insula |
|  |  |
|  | 4000 wm-rh-unknown |
|  | 4001 wm-rh-bankssts |
|  | 4002 wm-rh-caudalanteriorcingulate |
|  | 4003 wm-rh-caudalmiddlefrontal |
|  | 4004 wm-rh-corpuscallosum |
|  | 4005 wm-rh-cuneus |
|  | 4006 wm-rh-entorhinal |
|  | 4007 wm-rh-fusiform |
|  | 4008 wm-rh-inferiorparietal |
|  | 4009 wm-rh-inferiortemporal |
|  | 4010 wm-rh-isthmuscingulate |
|  | 4011 wm-rh-lateraloccipital |
|  | 4012 wm-rh-lateralorbitofrontal |
|  | 4013 wm-rh-lingual |
|  | 4014 wm-rh-medialorbitofrontal |
|  | 4015 wm-rh-middletemporal |
|  | 4016 wm-rh-parahippocampal |
|  | 4017 wm-rh-paracentral |
|  | 4018 wm-rh-parsopercularis |
|  | 4019 wm-rh-parsorbitalis |
|  | 4020 wm-rh-parstriangularis |
|  | 4021 wm-rh-pericalcarine |
|  | 4022 wm-rh-postcentral |
|  | 4023 wm-rh-posteriorcingulate |
|  | 4024 wm-rh-precentral |
|  | 4025 wm-rh-precuneus |
|  | 4026 wm-rh-rostralanteriorcingulate |
|  | 4027 wm-rh-rostralmiddlefrontal |
|  | 4028 wm-rh-superiorfrontal |
|  | 4029 wm-rh-superiorparietal |
|  | 4030 wm-rh-superiortemporal |
|  | 4031 wm-rh-supramarginal |
|  | 4032 wm-rh-frontalpole |
|  | 4033 wm-rh-temporalpole |
|  | 4034 wm-rh-transversetemporal |
|  | 4035 wm-rh-insula |

Supplementary Table 3: Freesurfer ROIs, the systematically bad performing ROIs are marked in red

|  | GE 40 mT/m | Siemens 80 mT/m | Siemens 300 mT/m | |
| --- | --- | --- | --- | --- |
| **Subject** | **Standard (ST)** | **State-of-the-art (SA)** | **Standard (ST)** | **State-of-the-art (SA)** |
| H | 0.0103 | 0.0100 | 0.0081 | 0.0217 |
| L | 0.0164 | 0.0106 | 0.0070 | 0.0193 |
| M | 0.0136 | 0.0072 | 0.0095 | 0.0147 |
| N | 0.0137 | 0.0066 | 0.0062 | 0.0143 |

Supplementary Table 4a: MSE current registration

|  | GE 40 mT/m | Siemens 80 mT/m | Siemens 300 mT/m | |
| --- | --- | --- | --- | --- |
| **Subject** | **Standard (ST)** | **State-of-the-art (SA)** | **Standard (ST)** | **State-of-the-art (SA)** |
| H | 0.0050 | 0.0044 | 0.0053 | 0.0084 |
| L | 0.0056 | 0.0051 | 0.0041 | 0.0089 |
| M | 0.0065 | 0.0049 | 0.0080 | 0.0087 |
| N | 0.0047 | 0.0040 | 0.0049 | 0.0068 |

Supplementary Table 4b: MSE full nonlinear registration

|  | GE 40 mT/m | Siemens 80 mT/m | Siemens 300 mT/m | |
| --- | --- | --- | --- | --- |
| **Subject** | **Standard (ST)** | **State-of-the-art (SA)** | **Standard (ST)** | **State-of-the-art (SA)** |
| H | 1.0312 (0.2304) | 1.0153 (0.1470) | 0.9706 (0.1422) | 1.0357 (0.2084) |
| L | 1.0651 (0.2536) | 1.0297 (0.1949) | 0.9608 (0.1530) | 1.0412 (0.2285) |
| M | 1.0396 (0.2126) | 1.0153 (0.1400) | 0.9699 (0.1561) | 1.0223 (0.1819) |
| N | 1.0196 (0.2032) | 1.0261 (0.1570) | 0.9582 (0.1479) | 1.0394 (0.1840) |

Supplementary Table 4c: Mean (std) Jacobian determinant of nonlinear registration

Supplementary Material 4d: Animated gifs that show the original and full nonlinear registration for subject N.
